# Epidemiology of intestinal helminthiasis among school children with emphasis on *Schistosoma mansoni* infection in Wolaita zone, Southern Ethiopia

**DOI:** 10.1186/s12889-017-4499-x

**Published:** 2017-06-20

**Authors:** Bereket Alemayehu, Zewdneh Tomass, Fiseha Wadilo, Dawit Leja, Song Liang, Berhanu Erko

**Affiliations:** 1 Biomedical Sciences Division, Department of Biology, College of Natural and Computational Sciences, Wolaita Sodo University, Wolaita Sodo, Ethiopia; 2 Microbiology Unit, College of Health Sciences and Medicine, Wolaita Sodo University, Wolaita Sodo, Ethiopia; 3 Department of Environmental and Global Health, College of Public Health and Health Professions, University of Florida, Gainesville, FL 32610 USA; 4 Aklilu Lemma Institute of Pathobiology, Addis Ababa University, Addis Ababa, Ethiopia

**Keywords:** *Schistosoma mansoni*, *Biomphalaria Pfeifferi*, Intestinal helminths, Epidemiology, Endemicity, School children, Wolaita zone, Ethiopia

## Abstract

**Background:**

Intestinal helminth infections are major parasitic diseases causing public health problems in Ethiopia. Although the epidemiology of these infections are well documented in Ethiopia, new transmission foci for schistosomiasis are being reported in different parts of the country. The objective of this study was to assess the prevalence of *Schistosoma mansoni* and other intestinal helminth infections among school children and determine the endemicity of schistosomiasis in Wolaita Zone, southern Ethiopia.

**Methods:**

Cross-sectional parasitological and malacological surveys were conducted by collecting stool samples for microscopic examination and snails for intermediate host identification. Stool samples were collected from 503 children and processed for microscopic examination using Kato-Katz and formalin-ether concentration methods. Snails collected from aquatic environments in the study area were identified to species level and *Biomphalaria pfeifferi* snails, the intermediate host of *S. mansoni*,, were individually exposed to artificial light in order to induce cercariae shedding. Cercariae shed from snails were used to infect laboratory-bred Swiss albino mice in order to identify the schistosome to species level.

**Results:**

The overall prevalence of intestinal helminth infections was 72.2% among school children. *S. mansoni* infection prevalence was 58.6%. The prevalence and intensity of *S. mansoni* infections varied among schools and sex of children. Swimming was the only factor reported to be significantly associated with *S. mansoni* infection (AOR = 2.954, 95% CI:1.962-4.449). Other intestinal helminth species identified were hookworms (27.6%), *Ascaris lumbricoides* (8.7%), *E. vermicularis* (2.8%), *Taenia* species (2.6%), *T. trichiura* (1.2%) *and H. nana* (0.6%). Only *B. pfeifferi* snails collected from streams shed schistosome cercariae and 792 adult *S. mansoni* worms were harvested from mice exposed to cercariae shed from *B. pfeifferi* on the 6th week post-exposure.

**Conclusion:**

The present study found high level of intestinal helminth infections in the study area. The study also confirmed autochthonous transmission and endemicity of *S. mansoni* as evidenced by both parasitological and malacological findings as well as by further establishing infections in lab-bred mice. Therefore, there is a need to include the area in the control programs with anti-helminth drugs and also consider other complementary measures including sanitation, provision of clean water supply, and snail control.

## Background

Intestinal helminth infections are among the most common infections worldwide. Globally 4.5 billion people are estimated at risk and more than 2 million are ill as a result of soil-transmitted helminths (STHs) infections in areas where sanitation is inadequate and water supplies are unsafe [1].

Schistosomiasis is also a common helminthiasis affecting more than 200 million people globally [1]. The global disease burden caused by schistosomiasis alone is estimated at 4.5 million disability-adjusted life years (DALYs) [2]. The disease is endemic in many countries of the world with over 90% of cases occurring in sub-Saharan Africa [3, 4]. Among the world population, it has been estimated that approximately 779 million people are at risk of schistosome infection [5–7].

Schistosomiasis is one of the major public health problems in Ethiopia. The two species of schistosome causing the disease in the country are *S. mansoni* and *S. haematobium*. Infections by the parasites usually occur in agricultural communities along small streams, irrigation schemes and lakes at altitudes ranging from 1300 to 2000 m above sea level (a.s.l) for *S. mansoni* and below 800 m a.s.l for *S. haematobium* [8–10]. Regarding distribution of the two schistosome species in Ethiopia, *S. mansoni* is widely distributed and *S. haematobium* is confined to the lowland areas of the country [9].

Presence of humans carrying the parasites and fresh water bodies harboring the snail intermediate hosts coupled with the suitable climatic conditions contribute to endemicity of both *S. mansoni* and *S. haematobium* infections in several localities in Ethiopia [9–16]. Furthermore, population movement and expansion of water resource development projects are playing key roles in establishing the parasite transmission in new areas in the country [9–11]. In Ethiopia, *S. mansoni* is transmitted by *B. pfeifferi* and *B. sudanica* while *S. haematobium* is transmitted by *Bulinus abyssinicus* and *Bu. africanus* snail intermediate hosts. *B. pfeifferi* has a wider geographical distribution, whereas *B. sudanica* is distributed in few low land areas of Ethiopia [17, 18]. Even though about 10 bulinid species are reported to exist in Ethiopia, only *Bu. abyssinicus* and *Bu. africanus* are found naturally transmitting *S. haematobium* in the country [15]. It has been reported in previous studies that *Bu. Abyssinicus* serves as the primary intermediate host for *S. heamatobium* in Awash valley, whereas *Bu. africanus* is dominant in Kurmuk (an area located near to Sudan) [9].

Although several studies have been conducted on the distribution and prevalence of intestinal helminthiasis and schistosomiasis in other parts of Ethiopia [9–19], only limited work was done to determine the prevalence and associated risk factors for these infections in rural villages in the present study area [19]. No epidemiological studies focusing on the parasite and intermediate hosts have been conducted in the area to determine the establishment of *S. mansoni* transmission. Therefore, the present epidemiological study aimed to assess the prevalence of *S. mansoni* and other intestinal helminth infections among school children and determine the establishment of *S. mansoni* transmission in the study area by conducting parasitological and malacological techniques as well as by establishing infections in lab-bred mice.

## Methods

### Study areas

The study was conducted in Wolaita Zone, located in the Southern Nations Nationalities and Peoples Region (SNNPR), Ethiopia. Wolaita Zone is about 329 kms south of Addis Ababa, Ethiopia. The Zone covers a total area of 4541 km^2^ and has an estimated population of 1.7 million [20]. Administratively, the Zone is divided into 12 districts and 3 town administrations. This Zone is one of the most densely populated Zones in the country with an average of 290 people per square kilometer. The majority of people living in the Zone earn their livelihood from subsistence farming [21]. Agro-ecologically, Wolaita is composed of 3 agro-ecological zones: 9% highland (“*dega*”), 56% midland temperate (“*woinadega*”), and 35% lowland (“*kolla*”), with altitude ranging from below 1000 m at the foot of Omo River Valley to 3000 m above sea level at Mount Damota. The average annual temperature varies from 15 °C to 30 °C, and the rainy season is bimodal with a shorter one starting from February to May and a longer rainy season lasting from June to August. The average annual rainfall of the Zone is 1350 mm. There are currently one governmental and two nongovernmental hospitals and eleven health centers in the zone.

### Study design and population

A cross-sectional study was carried out between January and February 2015 to determine the endemicity of *S. mansoni* infection in the study area, and assess the prevalence and associated risk factors of intestinal helminth infections among school children of five purposively selected primary schools of the rural *kebeles* (the smallest administrative units in Ethiopia) in close proximity to rivers and streams suspected as sources of schistosomiasis in the Zone. Purposive sampling of the schools was done based on schistosomiasis case information obtained from the health centers in the districts where the target primary schools are located.

School administrators were requested to provide students’ enrollment list. Students of grade 1-8 were selected because of their age limit that coincides with the standard age range (5-19 years) for schistosomiasis survey as set by World Health Organization (WHO). In each selected primary school, one classroom was randomly selected in each grade from grades 1 to 8. Then the study subjects were selected by systematic random sampling using their enrollment list as a sampling frame. Each selected subject was registered in a separate registration format and invited to participate in the study.

### Sample size determination

The sample size needed for this study was calculated using single proportion formula at 95% confidence interval (CI) level (Zα/2 = 1.96), assuming a previous prevalence of *S. mansoni* infection at 81.3%, as a baseline prevalence in the study area [19] and 5% marginal error. Then the sample size was calculated as n = (Zα/2)^2^ * P (1 – P)/ d^2^ [22], where n is minimum number of sample size, P is expected prevalence, Zα/2 is CI of 95%, and d is marginal error to be tolerated. To adjust for possible loss of effectiveness using the two-stage cluster sampling and obtain a comparable sample size, the design effect of 2 (assuming the sample variance is 2 time bigger than it would be under simple random sampling) was considered in the sample size estimation. By adding 10% for non-respondent rate, the final sample size was determined to be 515.

### Stool sample collection and examination

Stool samples were collected from school children who had no history of taking anti-helminthic drug in the past three months. Children who had no apparent chronic infections and were able to give stool samples were included in the study. Each study subject was given a plastic sheet with an applicator stick to bring approximately 4 g stool sample. A portion of the stool sample was used to prepare a single Kato-Katz slide in the field [23]. The remaining portion of the stool was transferred to stool cup and preserved in sodium acetate acetic acid formalin (SAF) solution for concentration technique [24]. Kato-Katz slides and the preserved stool samples were transported to the Biomedical Science Laboratory, Department of Biology, Wolaita Sodo University, for microscopic examination. A Kato-Katz slide prepared for each child was used for identification and egg count of *S. mansoni* and determination of the presence and absence of other intestinal helminths except for hookworms. Since a template delivering 41.7 mg of stool was used to prepare Kato-Katz slides, the egg of *S. mansoni* in the slide was counted and the number of eggs was multiplied by 24 to convert into eggs per gram of stool (EPG). The intensity of *S. mansoni* infection was calculated as the geometric mean for infected children and classified based on the intensity classes recommended by WHO as light (1–99 epg), moderate (100– 399 epg) and heavy (epg ≥ 400) [25].

### Snail survey and laboratory-mice infection

Snail intermediate hosts were surveyed in natural and man-made water bodies in the study areas. Decaying woods, plant remains, submerged vegetation and stones were searched for snails by trained field collectors using standard snail scoops or by hand picking using gloves and forceps. The same collectors scooped for snails in the surveys to standardize the sampling. Collected snails were placed in plastic buckets containing aquatic weeds and labeled for each collection site before transporting to Aklilu Lemma Institute of Pathobiology for identification, determination of infection by schistosome and maintaining the lifecycle of the parasite in mice. Snails were identified using shell morphology [26, 27].

*B. pfeifferi* snails were placed individually in shedding vials containing de-chlorinated water, and exposed to artificial light for about half an hour to induce shedding of cercariae. Laboratory-bred albino mice were exposed to cercariae for about 30 min by immersing in beakers containing cercariae infested water. Feces of mice were checked for the presence of schistosome eggs on day 43 post exposure. Mice were perfused [28] for harvesting of schistosome worms from blood vessels around the mesentery on day 45 post exposure.

### Data analysis

Statistical analysis was performed by using SPSS software version 17. Prevalence and intensity of *S. mansoni* infection were reported in percent and mean eggs per gram stool (EPG), respectively. Bivariate and multivariable logistic regressions were used to select candidate variables and determine the magnitude of association between *S. mansoni* infection and risk factors (age, sex, knowledge about schistosomiasis, herding cattle near river/stream, and reason for contact to water sources) by computing Odds Ratios (ORs) at 95% confidence level. All probability values were considered statistically significant when the calculated *P*-values were equal to or less than 0.05.

### Ethical considerations

The protocol of this study was reviewed and approved by the Ethical Review Committee of College of Natural and Computational Science in Wolaita Sodo University. Permissions were obtained from Wolaita Zone Health Department, District Health and Educational Bureaus to conduct the study. The objective of the study was explained to the school administration, primary health care providers and parents/guardian of the children. Verbal consent for the children was obtained from their parents/guardians. As most of the study population were illiterate, the ethical review committee endorsed oral consenting of the parents/guardians of the children. All children found positive for intestinal helminths were treated with appropriate drugs by nurses. The infection intensity and prevalence were reported to the Zonal and District Health Offices. The protocol for animal use was also reviewed and approved by the Ethical Review Committee of College of Natural and Computational Science in Wolaita Sodo University (Permit Number: EA-CNCS-07-R02). Handling of Swiss albino mice for sacrifice was performed under anesthesia, and all efforts were made to minimize suffering. The field study protocol of this research was also viewed and approved by the same ethical review committee.

## Results

### Socio-demographic characteristics and parasite infections

A total of 503 school children (287 males and 216 females) aged 5 to 19 participated in the present study with 98% response rate from five primary schools (Table 1). Seven different species of intestinal helminth parasites including *S. mansoni,* hookworms*, Ascaris lumbricoides, Trichuris trichiura, Taenia* species, *Hymenolepis nana* and *Enterobius vermicularis* were identified by both Kato-Katz and sodium acetate-acetic acid-formalin (SAF) concentration methods (Table 2). Among the school children examined for intestinal helminths, 72.2% were found positive for at least one of the helminth parasites identified. The highest prevalence of infection was due to *S. mansoni* (58.6%) followed by hookworm (27.6%) and *A. lumbricoides* (8.7%). Infections by *E. vermicularis* (2.8%), *Taenia* species (2.6%), *T. trichiura* (1.2%) and *H. nana* (0.6%) were less prevalent (Table 2). Among the five primary schools, the highest *S. mansoni* infection prevalence (75%) was observed in Gilo-Bisare, followed by 71% in Motala, 58.4% in Ajora, 40.6% in Bisare and 5.1% in Ello. Varying prevalence of major soil transmitted helminths was also observed among the schools (Table 3). Among the age categories, school children in the 5-9 and 10-14 years age group were highly infected. The prevalences of intestinal helminth infection by school were observed as 83.3% in Gilo-Bisare, 77.1% in Motala, 74.5% in Ajora, 46.9% in Bisare and 44.1% in Ello. Although there was no absolute dependence on a single water source for drinking and household consumption, pipe water pumped from the underground was the most (51.7%) utilized followed by the spring water (30%) and surface water (21.7%). 41.4% of children had either sometimes or never been informed about hygiene practices at home by their parents (Table 1).

**Table 1:** Prevalence of intestinal helminth infections with respect to socio-demographic characteristics of the study participants in Wolaita Zone, southern Ethiopia in 2015

Variables		Positive n(%)	Negative n(%)	Total n(%)
Sex	Male	215 (74.9)	72(25.1)	287
	Female	148 (68.5)	68 (31.5)	216
Age (Year)	5-9	66(74.2)	23 (25.8)	89
	10-14	269 (72.7)	101 (27.3)	370
	15-19	28 (63.6)	16 (36.4)	44
Schools	Ajora	111 (74.5)	38 (25.5)	149
	Gilo-Bisare	110 (83.3)	22 (16.7)	132
	Bisare	15 (46.9)	17 (53.1)	32
	Motala	101 (77.1)	30 (22.9)	131
	Ello	26 (44.1)	33 (55.9)	59
Source of drinking water	Pipe	194 (74.6)	66 (25.4)	260
	Wells	7 (87.5)	1 (12.5)	8
	Spring	117 (77.5)	34 (22.5)	151
	Surface water	69 (63.3)	40 (36.7)	109
Hygiene education by parents	No	15 (74)	5 (26)	20
	Sometimes	133(70.7)	55 (29.3)	188
	Regularly	215(72.9)	80 (27.1)	295

**Table 2:** Prevalence of intestinal helminth parasites in school children in Wolaita Zone, southern Ethiopia in 2015

Parasites	Positive	Negative	Prevalence
*Schistosoma mansoni*	295	208	58.6
*Ascaris lumbricoides*	44	459	8.7
Hookworms	139	364	27.6
*Trichuris trichiura*	6	497	1.2
*Taenia species*	13	490	2.6
*Hymenolepis nana*	3	500	0.6
*Enterobius vermicularis*	14	489	2.8
Overall infection	363	140	72.2

**Table 3:** Prevalence of *Schistosoma mansoni* and soil transmitted helminths (STH) infections (pooled result of Kato-Katz and SAF concentration methods) among school children in Wolaita Zone, southern Ethiopia in 2015

Helminth species	Age (Year)			Sex		School					Overall prevalence (%)
	5-9 (*n* = 89)	10-14 (*n* = 370)	15-19 (*n* = 44)	Male (*n* = 287)	Female (*n* = 216)	Ajora (*n* = 149)	Gilo-Bisare (*n* = 132)	Bisare (*n* = 32)	Motala (*n* = 131)	Ello (*n* = 59)	
*Schistosoma mansoni*	52 (58.4)	221 (59.7)	22 (50)	180 (62.7)	115 (53.2)	87 (58.4)	99 (75)	13 (40.6)	93 (71)	3 (5.1)	58.6
*Ascaris lumbricoides*	7 (7.9)	37 (10)	0	23 (8)	21 (9.7)	33 (22.1)	9 (6.8)	0	1 (0.8)	1 (1.7)	8.7
Hookworms	25 (28.1)	104 (28.1)	10 (22.7)	88 (30.7)	51 (23.6)	42(28.2)	38 (28.8)	0	36 (27.5)	23 (39)	27.6
*Trichuris trichiura*	0	4 (1.1)	2 (4.5)	4 (1.4)	2 (0.9)	1 (0.7)	2 (1.5)	0	2 (1.5)	1 (1.7)	1.2

### Intensity of *Schistosoma mansoni* infection

The overall geometric mean of faecal egg counts for *S. mansoni* was 189 EPG (ranged from 24 to 8736 EPG). Overall, 90 (34.2%) light, 94 (35.5%) moderate and 79 (30%) heavy intensity of *S. mansoni* infections among school children were observed. Among the age groups, the highest (24%) heavy intensity of infection was found in the 10-14 years age group. Males had more (16.7%) heavy infection intensity than females (13.3%). Among the schools, the highest heavy intensity of infection (15.6%) was observed in Motala followed by Gilo-Bisare (6.8%) and Ajora (6.5%). On the other hand, the light infections were reported for 34 children (12.9%) in Ajora, 27 (10.3%) in Gilo-Bisare, 25 (9.5%) in Motala, and 4 (1.5%) for Bisare primary schools (Table 4).

**Table 4:** Intensity of *Schistosoma mansoni* infection in school children in Wolaita Zones, southern Ethiopia, in 2015

Variables		Classes of intensity of infection^a^		
		Light n (%)	Moderate n (%)	Heavy n (%)
Age (Years)	5-9	17 (6.5)	14 (5.3)	12 (4.6)
	10-14	65 (24.7)	75 (28.5)	63 (24)
	15-19	8 (3)	5 (1.9)	4 (1.5)
Sex	Male	55 (20.9)	66 (25.1)	44 (16.7)
	Female	35 (13.3)	28 (10.6)	35 (13.3)
Schools	Ajora	34 (12.9)	32 (12.2)	17 (6.5)
	Gilo-Bisare	27 (10.3)	36 (13.7)	18 (6.8)
	Bisare	4 (1.5)	3 (1.1)	3 (1.1)
	Motala	25 (9.5)	23 (8.7)	41 (15.6)
Water contact frequency per week	≤ 2 days	27 (10.3)	22 (8.4)	29 (11)
	≥ 3 days	63 (24)	72 (27.4)	50 (19)
Reason for water contact	Swimming	77 (29.3)	78 (29.7)	56 (21.3)
	Other	13 (4.9)	16 (6.1)	23 (8.7)
Overall intensity	For total positive (*n* = 263)	90 (34.2)	94 (35.8)	79 (30)

^a^Classes of intensity for *S. mansoni* infection were set by WHO based on epg count as light (1–99 epg), moderate (100– 399 epg) and heavy (epg ≥ 400) [24]

### Risk factors associated with *S. mansoni* infection

Visual observations on droppings of human feces around water sources showed that people defecated in open field near rivers, streams and irrigation canals in the study area. It was also observed during visual field survey that human water contact activities in these study areas were also high. All of the school children visited the water bodies at least once in a week, either for swimming, washing clothes, bathing, or fetching water (Table 4). The multivariate logistic analysis showed that school children who swam in rivers and streams were 3 times more likely to be infected with *S. mansoni* than those who did not (AOR = 2.954, 95% CI:1.962-4.449) (Table 5). Although the AORs of some important variables such as sex and herding cattle near water bodies were not statistically significant, their COR showed significant difference (Table 5). There was no significant difference observed between *S. mansoni* infection and factors such as age and knowledge about the disease (*P* > 0.05) (Table 5).

**Table 5:** Association of *S. mansoni* infection with risk factors among school children in Wolaita Zones, southern Ethiopia in 2015

Variable		Positive	Negative	COR	95% C.I.		AOR	95% C.I.		*P*-value
					Lower	Upper		Lower	Upper	
Age	5-9	66	23	1.00			1.00			
	10-14	269	101	1.055	0.660	1.689	0.885	0.538	1.458	0.632
	15-19	28	16	0.712	0.344	1.470	0.656	0.306	1.404	0.277
Sex	Male	215	72	1.00			1.00			0.513
	Female	148	68	0.677	0.473	0.969	0.871	0.575	1.319	
Knowledge on disease	No	266	172	1.00			1.00			0.260
	Yes	29	36	0.521	0.308	0.881	0.722	0.410	1.272	
Herding cattle	No	113	97	1.00			1.00			0.398
	Yes	182	111	1.407	0.982	2.017	1.194	0.792	1.799	
Purpose of water contact	Other purpose	61	95	1.00			1.00			0.000
	Swimming	234	113	3.225	2.179	4.774	2.954*	1.962	4.449	

Variables adjusted: Age group, Sex, Know on disease, Herding cattle, purpose of water contact

*Significant association (*P* ≤ 0.05)

### Snail survey

Streams (Bisare and Kote), rivers (Woibo and Adacha) and Himbecho irrigation canal were surveyed for intermediate hosts during February and March 2015 and snails were collected using a scoop. Among the water bodies surveyed, Woibo and Adacha rivers harbored few and young *B. pfeifferi* attached to decaying wood, fallen leaves and stems of aquatic weeds.

A total of 256 snails (125 *Bulinus* species, 111 *B. pfeifferi* and 20 *Lymnaea natalensis*) were collected from Himbecho-Irrigation canal (15.6%), Kote (45.7%) and Bisare Streams (38.7%). *B. pfeifferi* collected from Kote and Bisare Streams shed schistosome cercariae. Ova were not observed in fecal materials from mice exposed to cercariae when checked at day 42 post exposure. Mice were sacrificed at day 45 post-exposure. A total of 792 schistosome worms were recuperated through perfusion of which 87 coupled *S. mansoni* adults were harvested from blood vessels around the mesentery of the mice (Table 6).

**Table 6:** Snails surveyed from water sources near human settlements in Wolaita Zone, southern Ethiopia, 2015

Variables		Water bodies			Total
		Himbecho Irrigation	Kote Stream	Bisare Stream	
Altitude (m.a.s.l)		1650	1802	1731	
Snail species	*Bulinus* species	18	67	40	125
	*Biomphalaria pfeifferi*	18	37	56	111
	*Lymnaea natalensis*	4	13	3	20
Total collection (%)		40 (15.6)	117 (45.7)	99 (38.7)	256
Number of *B. pfeifferi* shedding schistosome cercariae		0	5	2	7
Adult schistosome worms harvested after mice sacrificed	Male	0	527	83	610
	Female	0	0	8	8
	Copula	0	79	8	87

Observation on physical characteristics of the water bodies during the snail survey showed that Bisare and Kote streams were slightly turbid (having less suspended matter), and covered with abundant weeds, algae and other garbage droppings making the water environment conducive for snail existence. Both streams were relatively smaller and slow flowing as compared to the Woibo and Adacha Rivers. The muddy substratum of the streams favored the growth of both emergent and submerged plants.

## Discussion

Intestinal helminth infections are common public health problems in areas where sanitation is inadequate and water supplies are unsafe. Among these infections, soil transmitted helminthiasis due to *A. lumbricoides*, hookworms and *T. trichiura,* and schistosomiasis due to *S. haematobium* and *S. mansoni* are the most common in sub-Saharan Africa [29] These infections also remain considerable public health problems in Ethiopia and knowledge of high-risk localities is required to inform control interventions in such areas. Even though the mapping of schistosomiasis and soil-transmitted-helminthiasis have already been done in Ethiopia, continuous survey of the disease is needed to accommodate recent discoveries of new transmission foci possibly associated with expansion of water development projects and human movement in the country [14]. In line with this, there are recent reports on intestinal helminthiasis including schistosomiasis from the present study area [19, 30]. In the present study, the prevalence of intestinal helminth infections was determined and the endemicity of *S. mansoni* infection was established for the first time as evidenced by both parasitological and malacological surveys. The overall 72.3% prevalence of intestinal helminths including *S. mansoni* infections found in this study showed that intestinal helminth infections represent a considerable health problem in the study area.

In this study, varying magnitude of prevalence of infections by *S. mansoni,* hookworms*, A. lumbricoides, T. trichiura, Taenia* species, *H. nana* and *E. vermicularis* were found. These infections occurred in almost all of the areas under study. The distribution of these parasites over the study sites might reflect the existence of comparatively similar level of intestinal helminth infection condition in the area. Regarding STH infection, hookworms and *A. lumbricoides* were the major parasites identified in this study. The prevalence of hookworm infection (27.6%) found in this study is higher than the estimated national prevalence 16% [31] and this is in line with previous studies done in the country [30–37]. On the other hand, the prevalence of *A. lumbricoides* (8.9%) is quite lower than the estimated national prevalence 37% [31]. There are previous studies which reported higher prevalence of ascariasis in the country [37–40]. Infections by *E. vermicularis* (2.8%), *Taenia* species (2.6%), *T. trichiura* (1.2%) *and H. nana* (0.6%) were also recorded at low prevalence in the present study. Such variations in prevalence of helminth infections are likely attributed to variations in appropriate water supplies, sanitation, hygiene (WASH), among other things.

The prevalence of *S. mansoni* infections in 503 school children of 5 selected primary schools in Wolaita Zone, southern Ethiopia was found out to be 58.6%. This finding is lower than the previous 81.3% *S. mansoni infection* prevalence reported from one of the primary schools, Demba Girara, in a village of the present study area [19]. The lower prevalence in the present study might be due to variation of localities from where the school children were sampled. The current finding on *S. mansoni* infection prevalence was also lower than the 82.8% prevalence reported from Sanja area, northern Ethiopia [35] and 73.7% from Bushulo village, southern Ethiopia [36]. On the other hand, the current *S. mansoni* infection prevalence is higher than similar studies done on school children [37–42]. The higher *S. mansoni* infection prevalence in the present study might be due to the absence of clean water supply, lack of awareness on the disease and hence the absence of schistosomiasis control measures in the study area. Inhabitants of the area largely depended on open water sources for domestic and recreational use. The fact that inhabitants of the study area have no awareness on the existence of schistosomiasis manifested in that the disease was highly neglected in the area. Open defecation near water sources might have also increased the transmission of *S. mansoni* in the present study area.

The higher *S. mansoni* prevalence among male children might be due to the observed higher frequency of contact with cercariae contaminated water bodies than females. This finding is in agreement with previous studies done elsewhere in the country [19, 43–46]. The increased *S. mansoni* infection among male children could also be due to herding cattle near cercariae contaminated water bodies as this is an outdoor activity which is mostly practiced by male children in the study area. Similar explanation was given by the study done previously around the present study area [19]. The variation in *S. mansoni* infection prevalence (ranged 5.1% to 75%) among the primary schools might be due to the differences in altitude, microclimate and level of dependency in open water sources of the localities. The lower prevalence (5.1%) at Ello primary school might be due to relatively lower altitude (1400 m above sea level) of the area. In area with lower altitudes where the water environments experience relatively higher temperature which may affect survival of snail hosts and parasites [47].

All three classes of intensity of infection by *S. mansoni* were found in the present study with highest heavy infection intensities observed in the 10-14 years age group from age category, males from sex category, Motala from school category, those who had frequent water contact and those who swam in infectious water sources. The reason for finding higher heavy infection in these specified categories of children could be due to the frequent exposure to *S. mansoni* infection [18, 46, 48]. The age-specific association of infection intensity in current study is also in agreement with the finding of previous study done in a village close to the present study area [19]. The highest heavy infection intensity observed in Motala primary school might be attributed to the absolute dependency of inhabitants on Bisare stream which was highly infested with infected snails. The sanitary condition of water environment was also very poor as high fecal contamination was observed along the course of the stream in general and several human-water contact points in particular at Bisare.

The present study showed that the frequency of water contact was the most associated risk factor for *S. mansoni* infection in that school children who swam in rivers and streams were three times more likely to be infected with *S. mansoni* than those who did not swim. Other previous studies also found swimming to be a common risk factor for *S. mansoni* infection. Although herding cattle near streams and rivers was significantly associated risk factor to *S. mansoni* infection in the previous study done in a village of the study area [19], the present study found no statistically significant association with the activity.

*S. mansoni* endemicity speculated in earlier study [19] was confirmed by the present study through survey of snail intermediate host, shedding of *S. mansoni* cercaria from the *B. pfeifferi* snails and identification of the parasite in mice infection model. Survey for *B. pfeifferi* was conducted at water sources of areas where schistosomiasis is determined. The snail survey showed more abundant *B. pfeifferi* in Bisare and Kote streams than in Himbecho irrigation canal. Presence of less abundant *B. pfeifferi* in Himbecho irrigation canal might be due to sparse vegetation coverage of the canal. The slow water flow and abundant aquatic vegetation made Bisare and Kote streams favorable habitats for *B. pfeifferi* and other snails including *Bulinus* species and *Lymnaea natalensis* [16]. Even though Woibo and Adacha rivers were suspected for *S. mansoni* infection, only few young *B. pfeifferi* snails were observed along these rivers. The absence of adult *B. pfeifferi* snails could be due to the reason that the survey was made a week after unexpected heavy rainfall in the area due to the El Nino effect. Among the physical variables of water environment, substrate, vegetation density and water velocity are the strongest predictors of snail presence [49]. During the rains, snail occurrence and density are even more affected in relatively large streams and rivers [49]. In contrast to the high *S. mansoni* infection prevalence (58.6%) found in this study, a very low proportion of *B. pfeifferi* snails collected in the present survey were shedding *S. mansoni* cercariae. This finding is in agreement with other studies done in endemic areas with high transmission where few or none of the snail hosts collected shed any schistosome cercariae [50, 51]. In a previous study done in Msambweni area, coastal province of Kenya, where high prevalence of human schistosomiasis occurred, it was observed that very low proportion of snails were found shedding schistosome cercariae [52]. Therefore, the higher schistosomiasis transmission in an area may not always coincide with the greater proportion of host snails shedding schistosome cercariae. The possible explanation given for this unexpected low cercariae shedding from the snail intermediate hosts, as mentioned in previous studies, could be the variation in percentage of infected snail, focal nature of schistosomiasis, dispersed distribution of infected snails and seasonal differences [51, 52].

## Conclusion

The present study showed high level of intestinal helminth infections in the study area. The existence of schistosome infected *B. pfeifferi* and high prevalence of *S. mansoni* infection among school children confirm the endemicity of *S. mansoni* in the study areas. The high prevalence of intestinal helminth infections in general and the autochthonous transmission of *S. mansoni* infection in particular in the study area require implementation of preventive chemotherapy and also consideration of other complementary measures including sanitation, provision of clean water supply, and snail control.

## Abbreviations

EPG: Egg per gram of stool
STH: Soil-transmitted helminths
WHO: World Health Organization
